# Potential impact of invasive surgical procedures on primary tumor growth and metastasis

**DOI:** 10.1007/s10585-018-9896-8

**Published:** 2018-05-04

**Authors:** Maria Alieva, Jacco van Rheenen, Marike L. D. Broekman

**Affiliations:** 1 Princess Máxima Center for Pediatric Oncology, Uppsalalaan 8, 3584 CT Utrecht, The Netherlands; 2 Department of Molecular Pathology, Oncode Institute, Netherlands Cancer Institute, Plesmanlaan 121, 1066 CX Amsterdam, The Netherlands; 3 Department of Neurology & Neurosurgery, Brain Center Rudolf Magnus, University Medical Center Utrecht, Heidelberglaan 100, 3584 CX Utrecht, The Netherlands

**Keywords:** Tumor resection, Biopsy, Metastasis, Tumor cell behavior, Immune suppression, Wound healing

## Abstract

Surgical procedures such as tumor resection and biopsy are still the gold standard for diagnosis and (determination of) treatment of solid tumors, and are prognostically beneficial for patients. However, growing evidence suggests that even a minor surgical trauma can influence several (patho) physiological processes that might promote postoperative metastatic spread and tumor recurrence. Local effects include tumor seeding and a wound healing response that can promote tumor cell migration, proliferation, differentiation, extracellular matrix remodeling, angiogenesis and extravasation. In addition, local and systemic immunosuppression impairs antitumor immunity and contributes to tumor cell survival. Surgical manipulation of the tumor can result in cancer cell release into the circulation, thus increasing the chance of tumor cell dissemination. To prevent these undesired effects of surgical interventions, therapeutic strategies targeting immune response exacerbation or alteration have been proposed. This review summarizes the current literature regarding these local, systemic and secondary site effects of surgical interventions on tumor progression and dissemination, and discusses studies that aimed to identify potential therapeutic approaches to prevent these effects in order to further increase the clinical benefit from surgical procedures.

## Introduction

Tumor tissue resection and biopsy are still indispensable for diagnosis, cytoreduction and determination of subsequent treatment strategies of solid tumors and, importantly, to extend the patients’ lifespan or contribute to their cure. However, also a wide range of potential undesired effects have been associated with these procedures. Negative consequences of tumor invasive procedures not only include post-operative complications, such as hemorrhage, surgical site infections and venous-thromboembolic complications [1–3], but also changes affecting the remaining tumor cells, such as increased survival, proliferation and migration [4, 5]. An increasing body of evidence from clinical and experimental studies suggests that surgical trauma caused by biopsies or resections gives rise to a series of local and systemic events that can potentially promote tumor progression and metastatic disease [4, 6–12]. Surgical interventions disrupt the tumor and the surrounding tissue and result in tumor cell displacement to healthy tissue and release into the circulation. Moreover the inflicted surgical trauma initiates a wound healing response that activates a series of humoral and cellular cascades aimed at closing the wound [13]. While necessary for normal tissue repair, the local and systemic alteration of the innate and adaptive immune responses can stimulate tumor cell malignant behavior, survival, angiogenesis and extravasation of circulating tumor cells.

These undesired effects have the potential to affect tumor progression and spread. However, this does not imply that surgical resection or biopsy should be abandoned since these effects may only hold true for a subset of patients and the prognostic benefit of these procedures still strongly outweigh their negative effects. Nevertheless, it will be important to fully understand their impact on tumor pathology in order to prevent these effects and develop adjuvant treatments that would make these procedures even safer and more beneficial for the patients.

In this review, we summarize existing data that illustrates the effects of surgery on tumor progression and discuss potential strategies to circumvent these undesired effects of surgical procedures.

## Local effects of surgery

### Biopsies and tumor cell seeding

Fine needle aspirations (FNAs), in which a thin fine-gauge needle is inserted into the core of the tumor to aspirate tumor cells, are commonly performed to obtain tissue for diagnosis. This procedure has been associated not only with standard surgical complications as surgical site infections and hemorrhage, but also with a risk of tumor cell seeding along the needle track into the adjacent tissues. This risk is inherent to the procedure in which a needle transgresses and disrupts the tumor and is withdrawn. Histological evidence suggests that upon FNA tumor cells can be found in adjacent tissues [14–16]. However, some studies question the viability of the remaining tumor cells. For example, in breast tumors undergoing biopsy the incidence of tumor cell seeding in the needle track seemed to decline as the time between the biopsy and the subsequent resection increased, suggesting that the displaced cells rarely survive [17].

**Fig. 1:**
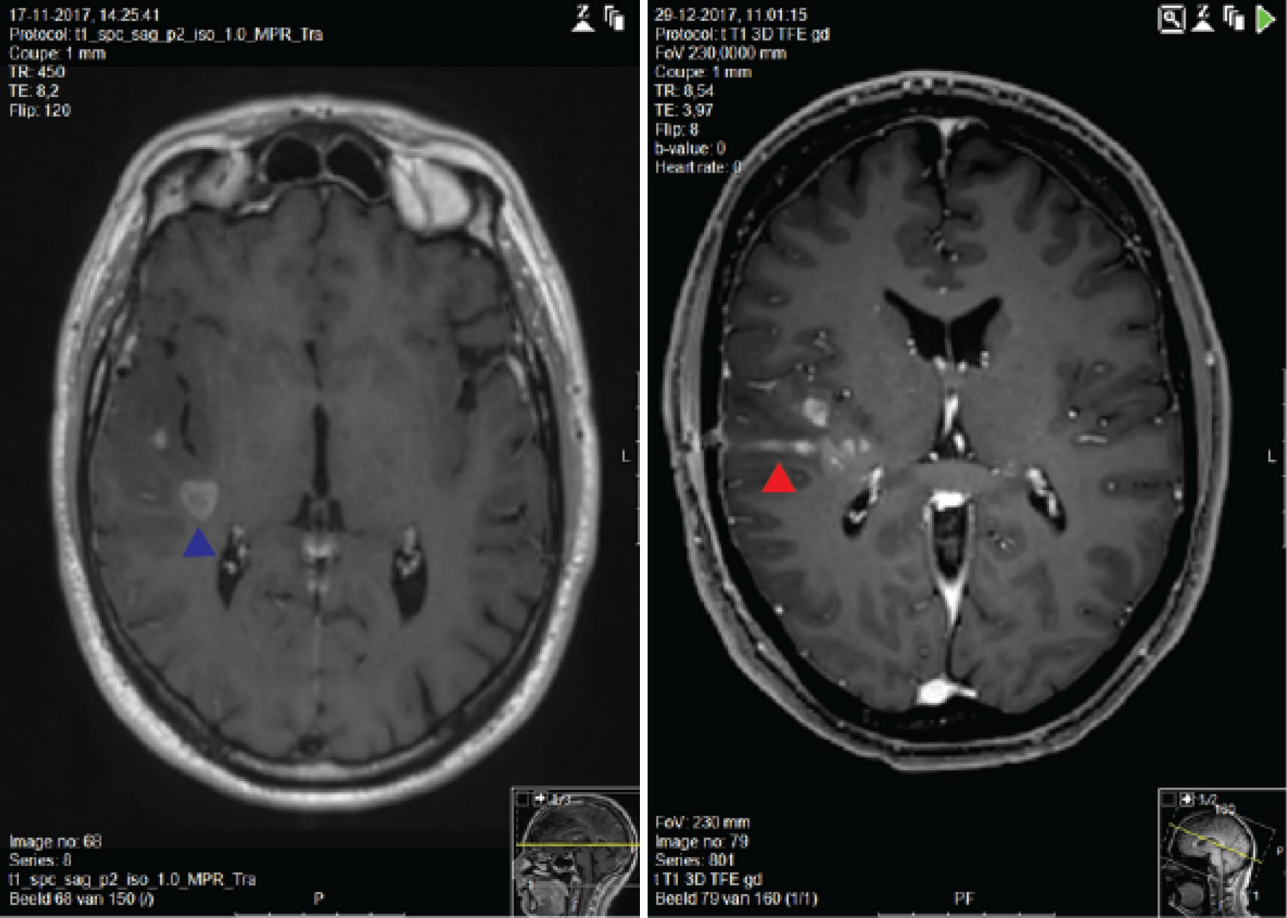
Evidence of tumor seeding after needle biopsy of a glioblastoma. Six weeks after a needle biopsy of a glioblastoma (blue arrow), MR-imaging shows evidence of tumor seeding along the needle track (red arrow). (Color figure online)

The use of larger needles has also been associated with tumor seeding into the adjacent tissues. For example in brain malignancies, tumor cells could be found in the needle track after biopsy of gliomas [18, 19] (Fig. 1) or of brain metastases [20, 21]. However, the incidence of viable tumors and tumor recurrences along the track is low and hard to quantify. For hepatocellular carcinoma, a recent meta-analysis of the available literature showed that, overall the pooled estimate of a patient with seeding per 100 patients is 0.027 and that, per year the pooled estimate of a patient with seeding per 100 patients is 0.009 [22]. For other cancers, where FNA is a common practice, such as thyroid cancer, needle track seeding is extremely rare and only a few cases have been reported [23]. Percutaneous FNA, performed on pancreatic tissue has also been associated with coelomic seeding [24]. However distinct studies present conflicting results on the incidence of seeding and its risk for tumor progression [25]. The use of endoscopic ultrasound-FNA allows a more accurate lesion sampling and thus may eliminate the risk of coelomic seeding [26]. The risk of tumor seeding after needle biopsy in retroperitoneal sarcoma is estimated to be 0.37%, based on pooled data from four tertiary care centers [27–29]. Even though the data is limited, few studies indicate that the use of vacuum assisted biopsy devices might reduce the risk of tumor seeding compared to the use of traditional devices [17, 30].

### Effect of tumor resections on tumor cell seeding

For many solid tumor types, resection is part of the treatment plan. Often, the aim of surgery is to completely resect a tumor (gross total resection), but partial resections are sometimes the only clinical option. Various tumors including chordoma [31] and mixed epithelial stromal tumor of the kidney [32] have also shown evidence of tumor seeding as a result of partial resections. Unfortunately, even gross total resection of tumors can result in local dissemination of cells. For example, it has been shown that piece meal resection of brain metastases increases the risk of leptomeningeal spread (LMS) resulting in worse outcomes for patients [33]. In addition, recent evidence suggests that the risk of LMS is higher after surgery followed by stereotactic radiosurgery (SRS) than after SRS alone [34].

Other examples of tumor cell dissemination after resection include port-site recurrences after laparoscopic abdominal oncological surgeries, procedures with clear advantages associated with their minimally invasive character [35]. Various factors seem to contribute to tumor seeding in this context. First, some factors might be tumor related. High-grade or high-stage tumors have been associated with a greater risk of seeding [36, 37]. Second, there might be wound related aspects that offer a tumor supportive environment. This process can contribute to tumor metastasis and will be discussed below. Third, there might be surgery-associated factors. For example, it has been suggested that the CO_2_ pneumoperitoneum plays a role in the development of these metastases as well as in the development of peritoneal carcinomatosis after laparoscopic surgery [38, 39]. Dry cold CO_2_ might induce peritoneal damage facilitating metastases [40, 41], for example by creating a local immune suppressive micro-environment. Additionally, extensive manipulation of the tumor, has been associated with a higher risk of port site metastasis [42, 43]. Finally, not using a retrieval bag might facilitate port site metastasis [44]. To prevent these metastases, studies that aim to improve safety of surgery, for instance by identifying alternatives for the cold CO_2_ insufflation, like those performed in mice by Carpentiri et al., are essential [45]. In addition, it is important that surgeons are aware of the impact of the techniques they use and that they are properly trained to minimize the risk of tumor seeding, especially since training seems to decrease the risk of these unwanted negative effects of surgery [46].

### Immune suppressive micro-environment

Next to effects on tumor cell seeding, surgical procedures induce changes in the local tumor microenvironment that can impact tumor cell gene expression and behavior. Tumors are largely infiltrated by immune cells that can exert both pro-tumoral and anti-tumoral effects. To escape immune surveillance exerted by anti-tumoral effectors, tumor cells often create an immunosuppressive microenvironment that favors tumor progression and metastatic spreading [47, 48]. Surgical procedures, as tumor resection and biopsy, can even further promote immunosuppressive infiltrates in the remaining tumor mass [49, 50].

Both the innate and the adaptive immune systems contribute to this immune suppressive environment. In an experimental model of lung carcinoma, tumors that had undergone partial tumor resection were found infiltrated with immunosuppressive alternatively-activated macrophages and regulatory T cells (Tregs), which prevented CD8 T lymphocytes recruitment to the tumor and contributed to faster tumor recurrence [50]. Infiltration of these immunosuppressive cells was stimulated by high levels of TGF-β and COX-2 found in the resection area [50]. A study of oral squamous cell carcinomas showed that the number of alternatively activated macrophages in the tumor correlates with the size of the surgical trauma, with animals undergoing tumor resection having higher numbers of alternatively activated macrophages than animals that underwent a biopsy [51]. Still, even small wounds to the tumor created by needle biopsy can rapidly promote pro-inflammatory factors such as S100A8, CXCL2, CCL3, and COX2 followed by a significant increase in the number of myeloid derived suppressor cells in the tumor [49]. Eosinophils that controversially have been reported as indicators of both favorable [52, 53] and poor prognosis [54, 55] have also been found to infiltrate the tumor after biopsy in breast cancer patients [56].

### Tumor cell transformation and changes in tumor cell behavior

In addition to creating an immunosuppressive environment, infiltrating immune cells in response to surgical trauma are a source of inflammatory cytokines, chemokines and growth factors. These factors are known for their ability to promote tumor cell proliferation, differentiation and migration [57]. Numerous studies have found that tumor wounding by either resection or by biopsies stimulates tumor growth [4, 5, 56, 58–61]. Interestingly, the impact of wounding on tumor growth is distinct between tumor subtypes. For instance, luminal A breast tumor showed more Ki67 upon core needle biopsy compared to luminal B-HER2-tumors [61].

Different mechanisms explaining the local recurrence of the tumor have been proposed. Increase in levels of VEGF and endostatin in the wounded tissue potentiate neoangiogenesis, required for tumor growth [62, 63]. Mitogens, such as heparin-binding epidermal growth factor, platelet-derived growth factor, TGF-β, basic fibroblast growth factor present in the wound fluid support tumor growth rate [58, 59]. These growth factors can be secreted directly by the tumor cells, due to changes in their transcriptome [49]; or by recruited [4] and resident cells present in the wounded microenvironment [60]. Surgical trauma caused by tumor resection of brain tumors promotes reactive astrogliosis, that can either directly induce tumor proliferation thought secretion of mitogens (e.g. SDF-1) [64] or either through the recruitment of inflammatory cells such as macrophages [65].

Mathenge et al. reported that tumor biopsy promotes epithelial to mesenchymal transition (EMT) related changes in gene expression in the remaining tumor cells via an increase in TGF-β and SOX-4 production [49]. This fundamental process in embryonic development plays a crucial role in tumor cell invasion. In glioma, surgical resection and biopsy have shown to promote tumor cell proliferation through transcriptome and secretome alterations of reactive astrocytes [60] and recruitment of pro-inflammatory monocytes to the operative site [4].

Besides increased growth rate, surgical trauma can also enhance tumor cells’ migratory capacity [4, 60, 66], a key process in tumor cell local and systemic dissemination [67, 68]. The transition from epithelial to mesenchymal state that tumor cells can undergo upon biopsy [49], is reported to be necessary for tumor cells migratory behavior [4, 69]. Tumor cell migration and invasion in the surrounding tissue can be promoted by soluble factors as a consequence of changes in local tumor microenvironment [60] and the inflammatory cell influx [4] to the wounded site. The effects on local tumor cell invasive capacity are of particular importance for highly aggressive tumors such as high grade gliomas, in which local invasion prevents complete tumor resection and has a direct impact on patient’s outcome [70, 71]. For instance, reactive astrocytosis [60] as a result of surgical resection can promote tumor cell invasion via the secretion of distinct paracrine factors (e.g. TGF-α, CXCL12, S1P, GDNF, MMP-2, MMP-9) [72–74].

The development of intravital microscopy (IVM) [75], a novel imaging technique that allows to visualize tumor cell behavior at single cell level in a living animal, has granted a deeper insight into the impact of small surgical interventions, such as biopsy, on tumor progression and determine the cellular mechanisms behind this process [4, 66]. Using IVM, we have previously shown that biopsy-like injury induces migration and proliferation of tumor cells via recruitment of monocytes and their differentiation to macrophages [4]. Over the last decades it has been extensively reported that tumor associated macrophages are able to promote tumor cell migration thought secretion of matrix-remodeling proteins, cytokines and chemokines (e.g. MMP-2, MMP-9, TNF-α, VEGF, TGF-β, EGF) [76–80]. Some of these factors and others can also stimulate tumor proliferation and survival (e.g. EGF, PDGF, TGF-β1, HGF and FGF-2) [79, 81, 82]. Another study using IVM has shown that next to their ability to invade the surrounding tissue, human glioma cells have been shown to repopulate the surgical lesion area through directed migration to the wounded site [66]. In some models, it has been shown that this recolonization is at least in part mediated by tumor microtubes that form a tumor cell network and contribute to tumor cell invasion, proliferation in unlesioned tumor and to radiotherapy resistance [83]. Upon surgery, the neighboring tumor cells are able to extend new tumor microtubes and direct newly formed tumor cell nuclei towards the lesioned area to repopulate it [66].

## Systemic effects of surgery

### Tumor cell release

With the advance of successful treatments that target primary tumor sites, metastatic disease management and prevention has become the main challenge for most solid tumor treatment strategies [84]. However, surgical trauma results in systemic and secondary site changes that might favor the formation of new metastases and potentiate the growth of pre-existent micrometastases.

Because tumor cell dissemination to distant organs occurs via shedding of circulatory tumor cells (CTCs) into the lymphatic and blood vasculature, high CTC numbers are closely associated with metastatic disease progression and survival [85, 86]. Numerous studies from different types of cancer have reported cancer cell release into the circulation as a result of biopsy and surgical resection [49, 87–94].

Even non-invasive medical interventions such as external tumor palpation have been shown to lead to increased numbers of CTCs during and immediately after the procedure in subcutaneous models of melanoma and breast cancer [94], suggesting that tumor cells release into the circulation is susceptible to external manipulation. However, CTCs release upon surgery and its impact on patients’ prognosis is still controversial, since some studies show no increase in CTC counts before and after surgery [95], while others find that CTC counts after surgery are not associated with recurrence free survival or overall survival [96].

Interestingly, a time-course experiment over several weeks performed by Juralti et al. found that CTC dynamics increased immediately after punch biopsy and stayed elevated for 6 weeks after the procedure [93], while complete tumor resection led to a decrease in CTC counts. This difference is likely to be due to the high efficiency of tumor cell resection that can be achieved in a subcutaneous pre-clinical model where the complete primary tumor can be eliminated, and thus the source of CTCs. However, in the clinic complete tumor resection can be very challenging, making partial or subtotal resections a reality [97–99]. In these cases the remaining tumor cells can be a source of CTCs. In line with this notion Juralti et al. found that incomplete tumor resection led to an increased number of CTCs [93].

The mechanisms of CTC mobilization in the blood upon surgical trauma have yet to be clarified. The immediate shedding of CTCs in the bloodstream indicates passive intravasation due to mechanical pressure and vascular collapse [100]. However in later stages the local inflammatory response, described above, could also impact tumor cell capacity for active intravasation via changes in tumor cell gene expression [49] or changes in the microenvironment, that favor tumor cell migration [4] and vascular permeability [101].

### Systemic inflammation potentiates tumor cell survival in circulation and extravasation

Next to CTC release, invasive surgical interventions trigger changes in systemic inflammation that can impact the CTCs ability to survive and extravasate. Extensive evidence has shown that surgical interventions lead to an imbalance of the innate and adaptive immune regulatory mechanisms and impair immune functions [102–105]. One of the effects of surgery is systemic immunosuppression of cells that participate in CTCs clearance [106, 107] and in the control of tumor cell dissemination [108, 109]: natural killers (NK) and phagocytic monocytes/macrophages. This process is triggered by the activation of the hypothalamic–pituitary–adrenal axis in response to surgical trauma stress [110, 111]. NK cell numbers and cytotoxic activity experience a rapid and prolonged (up to 10–30 days) decrease due to surgical interventions [112, 113]. Additionally, monocytes/macrophages phagocytic and chemotactic functions and antigen recognition mechanisms are compromised due to surgery [114, 115] and even as a result of anesthesia [116, 117]. Likewise, suppression of NKs and macrophages in a model of hepatic metastasis has shown to potentiate tumor cell uptake and outgrowth in the liver [107].

An increase in neutrophil counts in blood [118, 119] is triggered by surgical trauma. As a result, tumor cell survival, via NK cell suppression, is promoted and tumor cell extravasation is stimulated through IL-1 and matrix metalloproteases secretion [120]. Moreover, in response to injury neutrophils expel granule and nuclear constituents (including DNA and histones) mixed with a variety of cytokines, known as neutrophil extracellular traps (NETs) [121]. These structures sequester CTCs [122] from circulation and promote tumor cell invasion and metastatic outgrowth [123]. Post-operative NET formations in a cohort of patients with liver resection for metastatic colorectal cancer were associated with the extent of surgical resection and resulted in an increased risk of recurrence [124]. Indeed, NETs inhibition, described in a murine model of surgical stress, attenuates the development of metastasis triggered by the surgical intervention [124].

Another systemic effect of surgery consists of an acute inflammatory response mediated by the release of inflammatory cytokines into circulation [104]. TNF-α and IL-1 are some of the earliest and most potent systemic mediators of inflammation known to stimulate tumor cell adhesion [125, 126], invasion [127] and neoangiogenesis [128, 129] and potentiate metastasis formation [130, 131]. In addition to inflammatory cytokines, an increase in pro-angiogenic factors, such as VEGF and angiopoietin-2 in plasma has been reported after distinct surgical interventions [63, 132, 133].

In the peritoneal cavity tumor cell adhesion can be facilitated due to exposure of the extracellular matrix after mesothelial cell detachment in response to surgical trauma [134]. This mechanism of regulation of peritoneal inflammation [135] has been shown to lead to increased tumor cell adhesion in non-traumatized areas of the peritoneal cavity [134].

## Secondary site effects of surgery

### Metastatic burden can be potentiated by surgical interventions

Apart from supporting the establishment of new metastases from CTCs, systemic effects of surgical trauma can potentiate the progression of preexisting metastatic foci and contribute to the creation of so called pre-metastatic niches. Over the last decades, numerous clinical and preclinical reports strongly relate surgical interventions such as tumor resection and biopsy with an increased metastatic burden in different types of cancer [6–12]. Different aspects of surgical interventions at the primary site ultimately contribute to tumor outgrowth at distant sites, some of which, such as tumor cell shedding in circulation, extravasation, immune cell modulation and pro-tumoral effects of inflammatory cytokines, have been discussed above. Surgical interventions to the primary tumor also exert direct effects on distant tumors. For instance, biopsy mediated acute inflammatory response promotes an increase in lung metastases, associated with neutrophils recruitment to the metastatic site that could be controlled with anti-inflammatory treatment or IL-6 inhibition [10]. Recruited neutrophils induce metastatic progression thought activation of intracellular growth signaling pathways mediated by Toll-like receptor 9 and NETs [124]. Additionally, Al-Sahaf et al. showed that upon primary tumor resection lung metastasis acquired an invasive and proangiogenic phenotype and increased proliferation [6], indicating that humoral responses to surgical trauma can also directly alter secondary tumor gene expression. Some effects of surgical interventions on tumor progression seem to be unrelated to the primary tumor, but rather a direct consequence of a normal wound healing response. In line with this, a wound to a tumor-free tissue has been shown to elicit systemic factors that promote tumor angiogenesis [136] and proliferation [58].

The potentiation of metastatic progression can also be a consequence of an alteration in the inhibitory control exerted by the primary tumor. The primary tumor secretes factors that control the growth of secondary metastasis; and surgical resection of the primary tumor releases this inhibitory control [137–139]. Regulation of angiogenesis plays a major role in this process. It is thought that both pro-angiogenic factors and inhibitors are secreted into the circulation by primary tumors, however inhibitors are more stable and can exert anti-angiogenic effects on distant micrometastases. As a result of primary tumor removal, angiogenesis inhibitors levels drop, an angiogenic switch takes place at distant tumor sites and gives rise to tumor expansion. Studies done by Folkman have elucidated the molecular mechanisms responsible for this effect with angiostatin and endostatin as main regulators [137–139].

### Pre-metastatic niche formation as a consequence of surgery

Little is known about the effects of surgery on pre-metastatic niche formation. However, the systemic inflammation elicited in response to surgery could also contribute to the alteration of the microenvironment at distant sites and create pre-metastatic niches. In line with this idea hepatic ischemia–reperfusion injury has shown to increase circulating bone marrow-derived progenitor cells accompanied by a marked microvascular density in the liver that resulted in a higher incidence of liver metastases, upon tumor cell injection after injury [140]. Hypoxic conditions at the primary tumor site created by decreased tissue perfusion upon a surgical intervention are also likely to stimulate pre-metastatic niche formation at distant sites [141]. Conditioned medium derived from hypoxic mammary tumor cells has been reported to increase bone marrow-derived cell infiltration into the lung and rise the metastatic burden at this site [142]. Future studies should focus on elucidating the mechanisms that contribute to the formation of pre-metastatic niches in response to surgical interventions at the primary tumor site.

## Prevention of undesired tumor promoting effects of surgical interventions

**Fig. 2:**
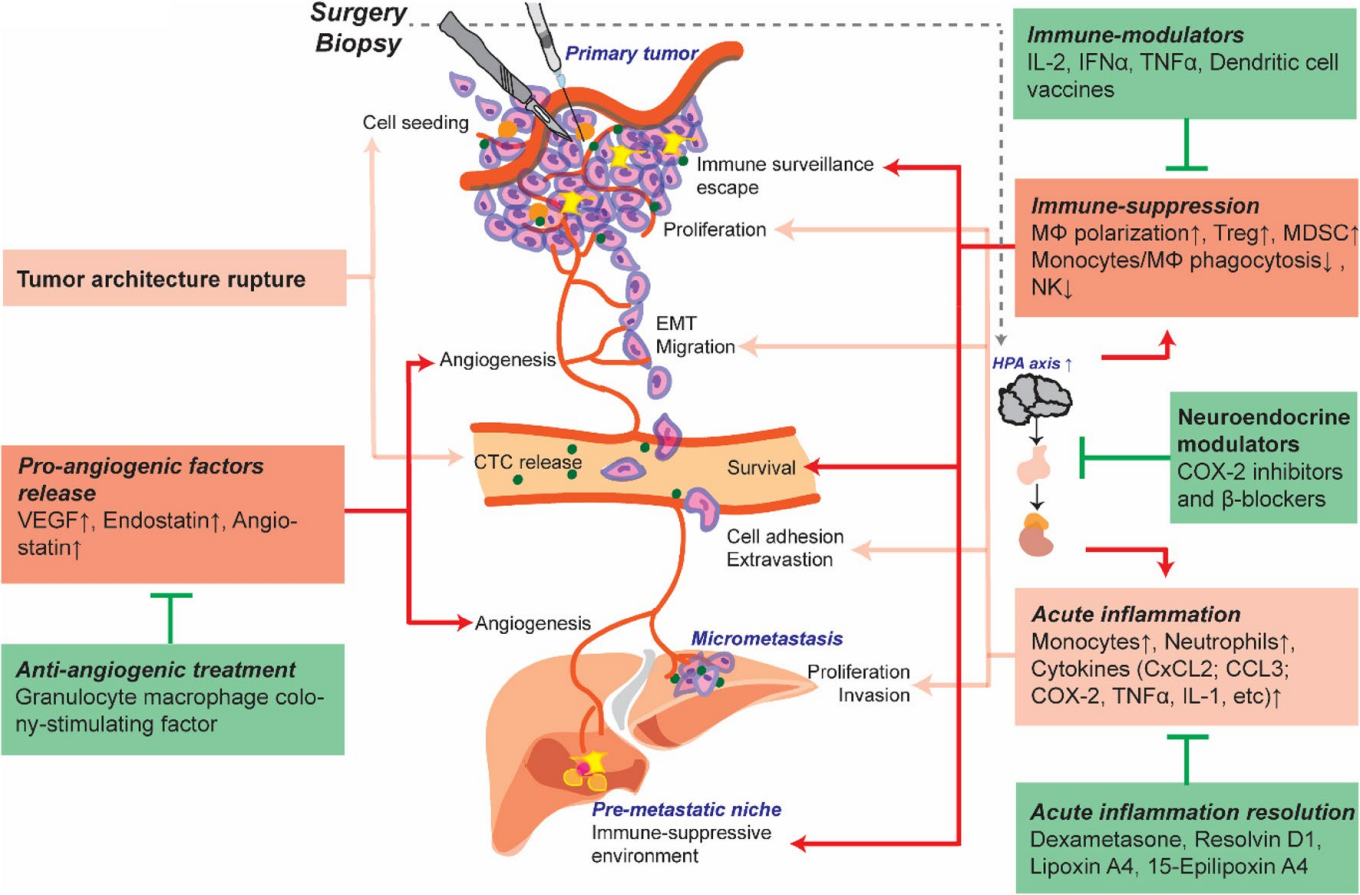
Surgical procedure impact on tumor cell progression and metastatic spread and prevention strategies. Surgical trauma induced immune-suppression, acute inflammation, pro-angiogenic factors release, and tumor architecture rupture contribute to tumor cell proliferation, migration, EMT; release and survival in circulation; adhesion to the endothelial wall and extravasation; escape of immune surveillance and angiogenic switch. Several therapeutic approaches show potential benefit to prevent these undesired effects: immune and neuroendocrine modulators, mediators of acute inflammation resolution and anti-angiogenic treatments. *IL-2* interleukin 2, *IFNα* interferon alpha, *TNFα* tumor necrosis factor alpha, *MΦ* macrophages, *Treg* regulatory T cell, *MDSC* myeloid-derived suppressor cell, *NK* natural killer, *COX-2* cyclooxygenase 2, *CxCL2* chemokine (C–X–C motif) ligand 2, *CCL3* chemokine (C–C motif) ligand 3, *IL-1* interleukin 1, *VEGF* vascular endothelial growth factor, *EMT* epithelial–mesenchymal transition, *CTC* circulating tumor cell, *HPA* hypothalamic–pituitary–adrenal

Extensive evidence links therapeutic and diagnostic invasive interventions with a stimulation of tumor progression. This does not imply however that the current cancer management protocols should be abandoned, as in most cases they allow for cure or extend lifespan of patients, that otherwise would not be possible. Instead the increasing knowledge of the cellular and molecular mechanisms underlying this phenomenon offer a therapeutic possibility to prevent these adverse effects, making clinical interventions even more beneficial for the patient. Several prevention strategies have been proposed (Fig. 2).

Since immune alteration plays a main role in surgery induced tumor progression perioperative immune-modulators have been proposed to suppress undesired effects of surgery. For example, IL-2 treatment prior to surgery has shown to counteract surgery-induced immunosuppression and extend survival of colorectal cancer patients [143, 144]. Other modulators such as granulocyte macrophage-colony stimulating factor, IFN-α and TNF-α also improved postoperative immune functions [130, 145, 146]. Although IL-2 and IFN-α treatments have shown severe side effects, some recent studies have reported that short-term administration schedules and drug delivery strategies induce only mild side-effects, suggesting that the use of these therapies should be further investigated to evaluate their potential to safely prevent surgery induced tumor progression [147, 148]. Another prospective strategy aimed to stimulate the adaptive immune response against the remaining tumor cells consists of the use of postoperative dendritic cell vaccines (DCV) [149, 150]. In the last years the optimization of DCV for therapeutic purposes has renewed the interest towards this approach and the number of clinical trials testing DCV is currently rising [151].

An alternative approach is to utilize factors that would resolve the acute inflammation that is triggered by surgical procedures [152]. Perioperative treatment with dexamethasone, a potent anti-inflammatory drug, has shown to prevent biopsy-induced tumor cell migration and proliferation by reducing monocytes recruitment to the injured site and reduced growth of biopsied tumors in multifocal glioblastoma patients [4]. Other mediators of inflammation resolution such as Resolvin D1, Lipoxin A4 [153] and 15-Epi-lipoxin A4 [154] represent a therapeutic potential due to their capacity to attenuate systemic inflammation.

Stress induced by surgery triggers the activation of the hypothalamic–pituitary–adrenal axis that modulates the immune system response via neuroendocrine factors [110, 111]. Preclinical studies have shown that perioperative blockade of neuroendocrine factors by COX-2 inhibitors and β-blockers can prevent immune suppression and decrease surgery-induced local tumor growth and metastatic progression [155, 156]. This notion has been supported by recent clinical data that report, not only the safe use of these treatments in patients, but also their efficacy to inhibit inflammatory ligands (IL-6, C-reactive protein), preserve the levels of cytokines that enhance anti-tumor activity by NK and cytotoxic T cells (IL-12, IFN-γ), decrease tumor infiltration with pro-inflammatory monocytes and abrogate the activity of immune suppressive CD4 T cells [157, 158]. Importantly, in the excised tumor preoperative treatment showed to suppress molecular pathways related to metastatic progression [157]. Although the impact of these treatments on long-term clinical outcome after surgery still needs to be assessed, the potential benefit of this approach is supported by studies that show association between the use of COX-2 inhibitors and β-blockers with progression-free survival in breast cancer patients [159, 160].

Tumor growth inhibition with preoperative [161, 162] or directly postoperative [163] chemotherapy or anti-angiogenic [164] treatments has previously been studied and although in some cases these therapies have shown to improve long term-survival [165] their use remains controversial. This is due not only to other studies where chemotherapy showed no benefit [166], but also because in some cases it seemed to elicit unwanted effects, similar to surgical trauma, that can contribute to tumor progression and spreading [167]. Moreover the adverse side effects of chemotherapy such as a strong inflammatory response and impaired wound healing constitute an additional challenge for their wide use in the clinic.

## Concluding remarks

Clinical and preclinical evidence indicate that both biopsies and tumor resections can have multiple effects that stimulate tumor progression, metastatic spreading and outgrowth. However, given the variety of surgical procedures, tumor types and stages that are encountered in the clinical practice, the conclusions drawn from these studies cannot be generalized. For instance, in early stage tumors, surgical procedures might have no impact on tumor progression because tumor cells still lack the genetic makeup necessary to acquire malignant features when a pro-inflammatory microenvironment is induced [61].

Moreover, the benefit of these surgical procedures for appropriate diagnosis and extended survival is undeniable and outweighs the potential negative effects, advocating against their discontinuation. Instead, the described findings show the importance of future studies addressed to further elucidate the cellular and molecular mechanisms behind these processes. This will allow to develop novel approaches to mitigate the local and systemic undesired effects of surgical interventions and even further improve the clinical benefit of these procedures.

## Abbreviations

FNAs: Fine needle aspirations
LMS: Leptomeningeal spread
SRS: Stereotactic radiosurgery
Tregs: Regulatory T cells
EMT: Epithelial to mesenchymal transition
CTCs: Circulatory tumor cells
NK: Natural killers
NETs: Neutrophil extracellular traps
HIF: Hypoxia inducible factors
RBCs: Red blood cells
